# The effect of non‐surgical periodontal treatment on progranulin levels

**DOI:** 10.1002/jper.11396

**Published:** 2025-08-25

**Authors:** Aysegul Sari, Pasquale Santamaria, Luigi Nibali

**Affiliations:** ^1^ Periodontology Unit, Centre for Host‐Microbiome Interactions King's College London Dental Institute London UK; ^2^ Faculty of Dentistry, Department of Periodontology Hatay Mustafa Kemal University Antakya Hatay Turkey

**Keywords:** non‐surgical periodontal therapy, periodontitis, progranulin

## Abstract

**Background:**

The aim of this study was to study the effect of non‐surgical periodontal therapy (NSPT) on gingival crevicular fluid (GCF) and serum progranulin (PGRN) levels in the early healing phases.

**Methods:**

The study included periodontitis (test) (*n* = 24) and periodontal health (control) (*n* = 24) groups. PGRN, vascular endothelial growth factor (VEGF), interleukin (IL)‐1β, tumor necrosis factor alpha (TNF‐α), and IL‐10 levels were assessed at baseline, at the 1st, 2nd, and 14th day, and 1st and 3rd month after NSPT in serum and GCF samples by Luminex bead‐based multiplex immunoassay method.

**Results:**

GCF PGRN, IL‐1β, TNF‐α, VEGF, and IL‐10 levels were higher in the test group than in the control group at baseline (*p* < 0.05). GCF PGRN and VEGF levels decreased from day 14 after NSPT, while IL‐1β levels decreased gradually from day 2 (*p* < 0.001). TNF‐α levels rapidly increased on day 1 after NSPT and gradually decreased from day 14 (*p* < 0.001). GCF PGRN/ TNF‐α molar ratio levels dramatically decreased from baseline day 1 after treatment and then increased gradually from day 14 to the 1st month (*p* < 0.001). There were no differences in serum parameters between groups and among time points (*p* ≥ 0.05), while a strong positive correlation was detected between GCF PGRN and IL‐1β, and TNF‐α levels (*p* < 0.001) at baseline.

**Conclusions:**

GCF PGRN total amount levels decreased gradually at each time point during the early healing period after NSPT, in parallel with IL‐1β. Changes in GCF PGRN and PGRN/TNF‐α molar ratio may be associated with periodontal disease and post‐treatment outcomes (ClinicalTrials.gov ID: NCT05535049).

**Plain language summary:**

Progranulin (PGRN) is a protein with complex physiological functions, producing granulin peptides that promote inflammatory and anti‐inflammatory activity. This study aimed to evaluate PGRN levels in the presence of periodontal disease and the local and systemic changes after following non‐surgical periodontal therapy (NSPT). The study included periodontitis (test) (*n* = 24) and periodontal health (control) (*n* = 24) groups. PGRN, vascular endothelial growth factor (VEGF), interleukin (IL) ‐1β, tumor necrosis factor alpha (TNF‐α), and IL‐10 levels were assessed in serum and gingival crevicular fluid (GCF) samples at baseline and at various time points after NSPT. GCF PGRN total amount levels were higher in the presence of periodontitis. Their levels decreased after NSPT from the 14th day in patients with periodontitis in parallel with inflammatory and regenerative mediators. However, PGRN/TNF‐α molar ratio levels increased after treatment at the end of the early healing phase. Molecular mediators hold promise as a diagnostic and therapeutic tool in periodontal therapy. Monitoring the level of PGRN in GCF before and after periodontal therapy could in the future be useful for personalized care.

## 1 INTRODUCTION

Periodontitis, a multifactorial chronic inflammatory disease, results from the interaction and development of dysbiotic communities and destructive inflammation.^1^ The complex cytokine network implicated in periodontitis encompasses specific cytokine receptors, anti‐inflammatory, and pro‐inflammatory cytokines. Cytokines in inflammatory periodontal tissues exert a pronounced influence on the onset and progression of periodontal disease.^2^ Host modulation therapies, such as anti‐cytokine therapy, can restore the balance between pro‐inflammatory and anti‐inflammatory mediators and create an environment that can reverse dysbiosis.^3^ Cytokine studies are being conducted to elucidate the role of cytokines in the immunopathology of periodontitis and to support host modulation therapies.

Nonsurgical periodontal therapy (NSPT) is the first‐line treatment for periodontal diseases and provides clinical improvements for clinical attachment levels (CAL) and probing pocket depth (PPD).^4^ Gingival crevicular fluid (GCF) is an exudate found in the gingival crevicular and may exhibit inflammatory characteristics.^5^ The role of a mediator in the inflammation or regeneration stages can be evaluated by GCF analyses after NSPT.^6^ Since processes such as inflammation, wound healing, and regeneration or repair after periodontal treatment involve a wide variety of molecules with multiple functions, the use of multiplex immunoassays that can simultaneously detect these molecules and their interactions may be advantageous.^6^ After periodontal treatment, early time points are important to detect early healing and to evaluate changes in the levels of GCF mediators.^7^ The inflammatory and early granulation tissue formation phase of wound healing lasts approximately 2 weeks after periodontal treatment. To follow the healing in the later period, a period of up to 3 months is a suitable time point.^6^ Additionally, serum mediators can be evaluated to assess the systemic effects of periodontitis and its treatment.^8^

Tumor necrosis factor alpha (TNF‐α) is a pro‐inflammatory cytokine thought to play a key role in the pathogenesis of periodontitis, with inhibitory effects on the osteogenic differentiation of periodontal ligament stem cells and bone regeneration.^9^ Progranulin (PGRN) is a glycosylated protein found in various tissues and is known as a granulin precursor.^10^ PGRN, also known as GRN‐epitelin precursor, is a growth factor found in adipocytes, immune cells, neurons, and epithelial cells.^11^ PGRN plays a critical role in pathophysiological processes such as tumorigenesis, early embryogenesis, neurodegeneration, and tissue repair.^12^ PGRN, which can act as an antagonist of TNF receptors (TNFRs), can reverse TNF‐α–mediated inhibition of osteoblast differentiation due to TNFR2 and promote bone regeneration.^13^ Studies on the role of PGRN in periodontal diseases are limited. In‐vitro and pre‐clinical studies focused on the effect of PGRN on bone regeneration in periodontal disease through promoting macrophage M2, activating TNF receptor 2 and (TNFR2) and ERK signaling, inhibiting TNF‐α and M1 macrophage polarization.^14^, ^15^, ^16^, ^17^ On the other hand, few human studies have shown that PGRN levels increase in the presence of periodontitis.^18^, ^19^, ^20^

It is still unclear whether PGRN plays a role in the inflammatory or repair/regenerative processes in periodontitis. To determine the role of PGRN in periodontitis, longitudinal human studies with are needed. To our knowledge, there is no study in the literature investigating PGRN levels after NSPT in the early phases of the healing process. Therefore, the primary aim of this investigation was to evaluate the GCF and serum PGRN levels after NSPT in the early phase and compare them in periodontitis and periodontal health, while the secondary aim was to correlate them with other pro‐inflammatory and repair/regenerative mediators. The null hypothesis of the study was that PGRN showed a similar pattern after NSPT at different time points and in periodontitis and periodontal health.

## 2 MATERIALS AND METHODS

### 2.1 Study population

The prospective cohort study was designed to characterize progranulin changes over time after non‐surgical periodontal treatment in serum and GCF. The prospective cohort study was carried out as a collaboration between King's College London Dental Institute, Periodontology Unit, Centre for Host‐Microbiome Interactions, United Kingdom, and Hatay Mustafa Kemal University, Turkey. The first step (clinical) of the study was carried out in Turkey, and the second (laboratory) step was in the United Kingdom. The study protocol was approved by the Ethics Committee for the Use of Human Subjects in Research of Hatay Mustafa Kemal University (Protocol No: 2022/90). The study was carried out in accordance with the tenets of the Declaration of Helsinki and conducted in the STROBE guidelines.^21^ The trial was registered on ClinicalTrials.gov (ID: NCT05535049). Individuals were included in the study from December 2019 through August 2021. Written informed consent was obtained from each participant before the clinical periodontal examination.

Forty‐eight adult individuals were enrolled in the study. Participants were recruited into 2 sex‐matched groups with 24 participants in each: periodontally healthy (PH) and periodontitis (P) groups. In accordance with the demographic variables of the patients included in the P group, the patients in the PH group were included.

The inclusion criteria for all participants included the following: (a) never smokers; (b) no history of periodontal treatment in the past 6 months; (c) no antibiotic therapy in the past 3 months; (d) more than 18 teeth present. The exclusion criteria included: (a) current pregnancy; (b) self‐reported systemic condition or disease, potentially affecting periodontal conditions, for example, AIDS, rheumatoid arthritis, cardiovascular diseases, and diabetes.

### 2.2 Periodontal parameters

Periodontal clinical parameters were recorded by a single calibrated examiner (intraclass correlation coefficient = 0.95) (author A.S.). The intra‐examiner reproducibility was determined for CAL, through repeated examinations of 10 subjects within a 1‐hour interval. Clinical periodontal measurements were assessed using the following periodontal measurements for periodontal diagnosis in both groups, and also at the 1st and 3rd month after non‐surgical periodontal treatment in the periodontitis group. The measurements were performed using a Williams periodontal probe (Hu‐Friedy, Chicago, IL, USA) and included PPD, CAL, plaque index (PI),^22^ gingival index (GI),^23^ and percentage bleeding on probing (BOP)^24^ at 6 sites per tooth (mesio‐buccal, buccal, disto‐buccal, mesio‐lingual, lingual, and disto‐lingual) on each tooth.

Diagnosis of periodontal diseases and conditions was made according to the radiographic and clinical diagnostic criteria proposed by the 2017 World Workshop on Classification of Periodontal and Peri‐implant Diseases and Conditions.^25^ Individuals with a BOP < 10% without attachment loss and radiographic bone loss were considered to have periodontal health.^26^ The criteria for periodontitis included patients with CAL ≥ 5 mm in 2 or more interproximal sites and PPD ≥ 6 mm in 1 or more interproximal sites. Only stage III–IV (severe) periodontitis was included in the present study.^27^

### 2.3 Periodontal intervention

Periodontitis patients were treated by 1‐stage full‐mouth disinfection, including full‐month scaling and subgingival instrumentation procedures within 24 h in 2 sessions. Each session was practiced for 60 min on 2 consecutive days. Subgingival irrigation and tongue brushing were made with 1% CHX gel for 1 min. Mouthwashes with 0.12% CHX were performed for 30 s at the beginning and the end of each session and twice a day for 2 weeks.^28^

### 2.4 Collection of GCF samples

GCF samples were collected following an 8‐h overnight fast and 1 week after clinical periodontal measurements at baseline in 2 groups and then at the 1st, 2nd, and 14th day, and 1st and 3rd month after NSPT in patients with periodontitis.^29^ The samples were collected from the site of teeth with PPDs ≥6 mm and BOP positive in patients with periodontitis, and from the site of teeth without attachment loss and radiographic bone loss and with BOP negative in healthy individuals. Three GCF samples were taken per participant. Samples were collected from a mesial or distal site on each of the 3 teeth (incisors, premolars, and molars). Saliva contamination was prevented by isolation with cotton rolls and gently air‐drying the sampling area. Samples contaminated with saliva or blood were not included. The plaque was gently removed from the sampling area by a curette. The samples were collected within 30 s with standardized paper strips (Periopaper; Oraflow Inc., Plainview, NY) by the orifice method.^30^ The volumes were measured on a pre‐calibrated electronic gingival fluid measuring device (Oraflow Inc., Plainview, NY)^||^.^31^ The values of the electronic device were referenced to a standard curve and converted to an actual volume (µL). All of the Periopaper strips were pooled in plastic Eppendorf microcentrifuge tubes. Samples were then stored at ‐80°C until analysis.

### 2.5 Collection of serum samples

Peripheral venous blood samples were taken at baseline from 2 groups and then at the 1st and 3rd month after NSPT from patients with periodontitis. The serum was separated from the cells by centrifugation at 2000 rpm for 10 min, after which it was stored at −80°C, until the biochemical analysis was performed.

### 2.6 Laboratory analyses

The samples of the patients were transferred to King's College London Dental Institute, Periodontology Unit, Centre for Host‐Microbiome Interactions, London, United Kingdom; after a material transfer agreement signed between Hatay Mustafa Kemal University, Hatay, Turkey and King's College Dental School, London, United Kingdom.

About 50 µL of phosphate buffered saline (PBS)/protease inhibitor cocktail (PBS with protease inhibitors 1X, Complete ULTRA tablets, Mini; ethylenediaminetetraacetic acid [EDTA]‐free) were added to a sterile microcentrifuge tube including 3 paper strips and centrifuged at 11,000 rpm for 15 min at 4°C for GCF elution. This procedure was repeated 2 times and obtained a total volume of 100 µL. They were stored at −80°C until the biochemical analysis.

Measurements of GCF and serum PGRN, VEGF, IL‐1β/IL‐1F2, TNF‐α, and IL‐10 levels were performed using a Luminex MAGPIX analyzer (Luminex; R&D systems). Luminex bead‐based multiplex immunoassay (Luminex, R&D systems, Minneapolis, MN) was used to carry out the analysis (in duplicate) of each sample. The assay included a series of known reference standards to generate logistic calibration curves, and all samples were quantified accordingly. The assay included a series of known reference standards to generate logistic calibration curves, and all samples were quantified accordingly. The mediator values were obtained as pg/mL.

The molar ratio was calculated between the levels of PGRN/TNF‐𝛼.

### 2.7 Statistical analysis

The main outcome of the study was GCF progranulin (ng/30s) levels after non‐surgical periodontal treatment in the healing phase. The study had originally been powered on periostin levels (study supported by Hatay Mustafa Kemal University as Project number: 19.M.051), resulting in 22 patients per group. For the current study, a post‐hoc power calculation was performed, based on the GCF PGRN total amount values. This calculation resulted in the need to recruit 24 patients in the periodontitis group to have 99% power for Cohen's *d* of 1.12 and 1.25 (for 14th day and 3rd month, respectively) and *α* = 0.05. Gpower package version 3.1 was used for power calculations.

The normality of the distribution of continuous variables was tested by Shapiro–Wilk test. Descriptive statistics were presented as mean and standard deviation (mean ± SD) for parametric distributed variables and median and interquartile range for the non‐parametric distributed variables.

Chi‐squared test was used to analyze the associations between categorical variables. Student *t* test for normally distributed numerical variables and Mann–Whitney *U* test for non‐normally distributed numerical variables were performed to compare 2 independent groups for numerical data. Friedman test was performed for different time points for non‐parametric variables. Post‐hoc pairwise comparisons were conducted using the Wilcoxon signed‐rank test with Bonferroni correction for multiple comparisons. Spearman's rank correlation coefficients were calculated to assess the correlations between numerical variables.

Statistical analysis was performed with SPSS for Windows version 29.0.2.0, and *p* values < 0.05 were accepted as statistically significant.

## 3 RESULTS

### 3.1 Demographic variables

Table 1 presents the demographic characteristics of the included participants. Sex, age, and body mass index (BMI) were similar between groups (*p* ≥ 0.05).

**TABLE 1:** Characteristics of the study population

Variables	Periodontal health group (*n* = 24)	Periodontitis group (*n* = 24)	*p*‐value
Sex			
Male	11 (45.8%)	11 (45.8%)	1
Female	13 (54.2%)	13 (54.2%)	
Age			
Mean ± SD	40.54 ± 9.07	41.54 ± 8.91	0.702
BMI			
Median (25%–75%)	24.22 (21.16–24.8)	24.76 (22.93–24.94)	0.264

*Note*: *P*‐value was obtained from chi squared test for categoric variables. *P*‐value was obtained from Student *t* test for parametric variables. *P*‐value was obtained from Mann–Whitney *U* test for non‐parametric variables. Statistically significant at *P* < 0.05.

Abbreviations: BMI, body mass index; SD, standard deviation.

### 3.2 Clinical periodontal findings

The periodontal parameters as full‐mouth and sampled teeth are shown in Table 2. Sampled teeth periodontal parameters and GCF values were the average values of 3 teeth. As expected, clinical periodontal parameters in periodontally healthy individuals were lower than the patients with periodontitis for both full‐month and sampled teeth (*p* = 0.001). All clinical periodontal parameters of full‐mouth and sampled teeth decreased from baseline to the 1st and the 3rd month (*p* = 0.001). There were no differences between the 1st and the 3rd month in PI and GI values of full‐mouth and sampled teeth and BOP values of sampled teeth (*p* ≥ 0.05). PD, CAL values of full‐mouth and sampled teeth, and full‐mouth BOP decreased from the 1st month to the 3rd month (*p* = 0.001). GCF volumes (uL) in periodontally healthy individuals were lower than in periodontitis patients (*p* = 0.001) and decreased gradually from baseline to the 3rd month (*p* = 0.001). There were no differences between the baseline and the 24th hour in GCF (uL) volume (*p* = 0.715).

**TABLE 2:** Full mouth and sampling clinical periodontal parameters between groups and among baseline and after treatment

Variables	Periodontal health group (*n* = 24)	Periodontitis group (*n* = 24)	*p*‐value^*^ (between groups)
PI (full mouth)			
Baseline	0.19 (0.05–0.62)	2.31 (1.44–3)	0.001
Month 1		0.05 (0–0.11)^†^	
Month 3		0.02 (0–0.11)^†^	
*p*‐value^**^ (among time points)		0.001	
GI (Full mouth)			
Baseline	0.17 (0.09–0.37)	2.24 (1.83–2.99)	0.001
Month 1		0.62 (0.29–0.93)^†^	
Month 3		0.29 (0.12–0.59)^†^	
*p* value^**^ (among times points)		0.001	
BOP (%) (Full mouth)			
Baseline	2.68 (0–7.14)	96.16 (82.18–100)	0.001
Month 1		17.78 (9.29–27.05)^†^	
Month 3		10.08 (5.06–14.02)^†^, ^¶^	
*p* value^**^ (among times points)		0.001	
PD mm (Full mouth)			
Baseline	0.46 (1.39–1.64)	4.43 (3.76–5.02)	0.001
Month 1		2.63 (2.15–3.41)^†^	
Month 3		1.98 (1.69–2.7)^†^, ^¶^	
*p* value^**^ (among times points)		0.001	
CAL mm (Full mouth)			
Baseline	0 (0–0)	4.65 (4.13–5.57)	0.001
Month 1		2.91 (2.54–3.41)^†^	
Month 3		2.08 (1.79–2.7) ^†^, ^¶^	
*p* value^**^ (among times points)		0.001	
No. of missing teeth number (Full mouth)			
Baseline	0 (0–0.67)	2 (1–5.5)	0.001
Month 1		3 (1–6)	
Month 3		3 (1–7)	
*p*‐value^**^ (among times points)		0.053	
PI (site)			
Baseline	0 (0–0.67)	2 (1.11–3)	0.001
Month 1		0 (0–0)^†^	
Month 3		0 (0–0)^†^	
*p*‐value^**^ (among time points)		0.001	
GI (site)			
Baseline	0 (0–0.11)	2.4 (2–3)	0.001
Month 1		0.55 (0.22–0.88)^†^	
Month 3		0.18 (0–0.44)^†^	
*p*‐value^**^ (among time points)		0.001	
BOP (%) (site)			
Baseline	0 (0–0)	100 (100–100)	0.001
Month 1		0 (0–0.22)^†^	
Month 3		0 (0–0.11)^†^	
*p*‐value^**^ (among time points)		0.001	
PD mm (site)			
Baseline	1.33 (1.22–1.7)	5.6 (4.95–6.7)	0.001
Month 1		3 (2.4–4)^†^	
Month 3		2.22 (1.8–3.1) ^†^, ^¶^	
*p*‐value^**^ (among time points)		0.001	
CAL mm (site)			
Baseline	0 (0–0)	5.9 (5.5–7.05)	0.001
Month 1		3.6 (3–4.3)^†^	
Month 3		2.9 (2.3–3.5)^†^, ^¶^	
*p*‐value^**^ (among time points)		0.001	
GCF ul (site)			
Baseline	0.1 (0.11–0.06)	0.79 (1.14–0.58)	0.001
24 h		0.88 (1.29–0.42)	
48 h		0.5 (0.74–0.34)^†^, ^‡^	
Day 14		0.2 (0.31–0.14) ^†^, ^‡^, ^||^	
Month 1		0.15 (0.23–0.09)^†^, ^‡^, ^||^, ^§^	
Month 3		0.1 (0.12–0.06)^†^, ^‡^, ^||^, ^§^	
*p*‐value^**^ (among time points)		0.001	

Abbreviations: BOP, bleeding on probing; CAL, clinical attachment level; GI, gingival index; PI, plaque index; PPD, probing pocket depth.

* *p*‐value was obtained from Mann–Whitney *U* test for between groups variables.

** *p*‐value was obtained from Friedman test for among times variables and Wilcoxon signed‐rank test for pairwise comparison. Data are expressed as median and interquartile range (IQR: 25–75). Statistically significant at *p* < 0.05

^†^ Versus baseline.

^‡^ Versus 24th h.

^||^ Versus 48th h.

^§^ Versus 14th day.

^¶^ Versus 1st month.

About 25% of patients required additional treatment after the 3‐month observation period.

### 3.3 Biochemical findings

The limit of detection (LOD) values for the mediators were as follows: 333.48 for PGRN, 2.02 for VEGF, 5.16 for IL‐1β/IL‐1F2, 1.97 for TNF‐α, and 1.27 for IL‐10 pg/mL. These values were accepted for the results under LOD. The under LOD values of all GCF and serum mediators ranged from 0 to 8.33% except: 25% for GCF PGRN at 3 month, 12.5%–29% for GCF TNF‐α at different time points, 12.5%–29% for GCF IL‐10 at different time points, 29% for serum TNF‐ in the periodontal health group at baseline.

#### 3.3.1 GCF

##### GCF total amount

**FIGURE 1:**
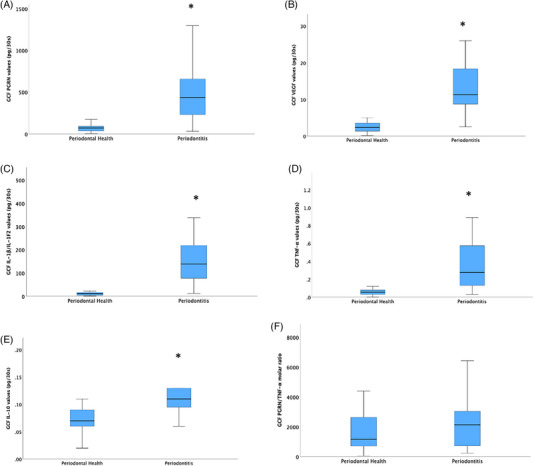
Gingival crevicular fluid mediators total amount levels at baseline between groups. (A) GCF PGRN values. (B) GCF VGEF values. (C) GCF IL‐1β/IL‐1F2 values. (D) GCF TNF‐α values. (E) GCF IL‐10 values. (F) GCF PGRN/TNF‐α values. *p*‐value was obtained from Mann–Whitney *U* test. Statistically significant at *p* < 0.05. *Versus periodontal health group. IL, interleukin; PGRN, progranulin; TNF‐α, tumor necrosis factor‐alpha; VEGF, vascular endothelial growth factor

**FIGURE 2:**
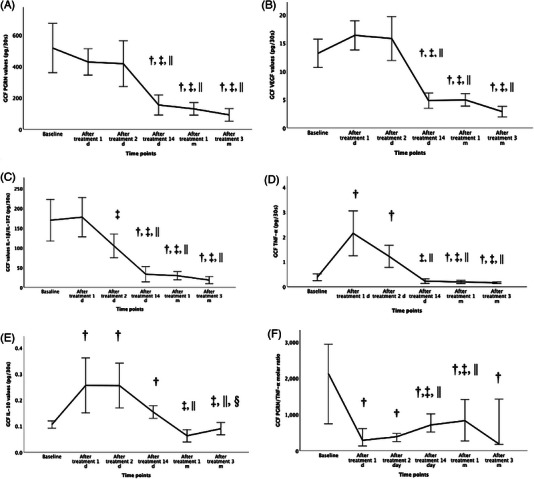
Gingival crevicular fluid mediators total amount levels baseline and after non‐surgical periodontal treatment time points. (A) GCF PGRN values. (B) GCF VGEF values. (C) GCF IL‐1β/IL‐1F2 values. (D) GCF TNF‐α values. (E) GCF IL‐10 values. (F) GCF PGRN/TNF‐α values. *p*‐value was obtained from Friedman test for among times variables and Wilcoxon signed‐rank test for pairwise comparison. Statistically significant at *p* < 0.05. † versus baseline, ‡ versus 24th h, || versus 48th h, § versus 14th day. IL, interleukin; PGRN, progranulin; TNF‐α, tumor necrosis factor‐alpha; VEGF, vascular endothelial growth factor

The GCF total amount levels are shown in Figures 1 and 2 (Table S1). GCF PGRN, IL‐1β/IL‐1F2, TNF‐α, VEGF, and IL‐10 total amount levels were higher in the test group than in the control group at baseline (*p* < 0.005), while there were no differences between groups in GCF PGRN/ TNF‐α molar ratio levels (*p* ≥ 0.05) (Figure 1). Figure 2 shows that short‐term statistically significant increases after NSPT (day 1) were detected only for TNF‐α and IL‐10 total GCF levels, with a decrease after day 2. A different pattern was detected for IL‐1 β/IL‐1F2 total amount levels, which decreased gradually from day 2 (*p* < 0.001), and PGRN and VEGF total amount levels, which started decreasing from day 14 after NSPT (*p* < 0.001). GCF PGRN/ TNF‐α molar ratio levels considerably decreased after treatment, from day 1 (*p* < 0.001), with a slight gradual increase from days 14 to 1 month (*p* < 0.001) (Figure 2).

##### GCF concentrations

The GCF concentration levels are shown in Table S1. Only TNF‐α and IL‐10 concentration values were statistically significantly higher in the periodontal health group than periodontitis group (*p* = 0.039 and <0.001, respectively). While PGRN concentration values did not change significantly during time points, VEGF concentrations increased after treatment, reaching a peak after 1 month (*p* = 0.001). IL‐1β/IL‐1F2 concentration decreased from day 14 (*p* = 0.012). GCF TNF‐α and IL‐10 concentrations showed a post‐NSPT increase up do day 14, similar to what was observed for total levels.

#### 3.3.2 Serum

**FIGURE 3:**
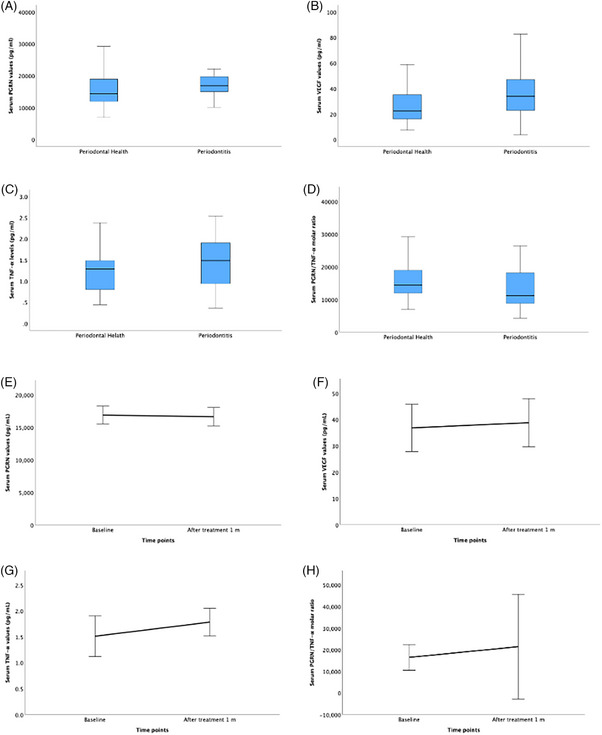
Serum mediators concentration values at baseline between groups and baseline and after non‐surgical periodontal treatment time points. (A) Serum PGRN values between groups. (B) Serum VGEF values between groups. (C) Serum TNF‐α values between groups. (D) PGRN/TNF‐α values between groups. (E) Serum PGRN values between time points. (F) Serum VGEF values between time points. (G) Serum TNF‐α values between time points. (H) PGRN/TNF‐α values between time points. *p*‐value was obtained from Mann–Whitney *U* test for between group variables. *p*‐value was obtained from Friedman test for among times variables and Wilcoxon signed‐rank test for pairwise comparison. Statistically significant at *p* < 0.05. IL, interleukin; PGRN, progranulin; TNF‐α, tumor necrosis factor‐alpha; VEGF, vascular endothelial growth factor

Serum biochemical parameters are shown in Figure 3 (Table S1). There were no differences in serum PGRN, VEGF, TNF‐α, and PGRN/ TNF‐α molar ratio levels between groups and between baseline and 1 month after NSPT (*p* ≥ 0.05). Their 3‐month values are not reported as the assay for that timepoint was not reliable. The IL‐1β/IL‐1F2 and IL‐10 results were out of range. Thus, the serum results of these mediators were not recorded.

#### 3.3.3 Correlations between GCF mediators and clinical periodontal parameters

Correlations between the total amount of GCF mediators and clinical periodontal parameters are shown in Table 3. There were strong positive correlations between PGRN and VEGF (*p* < 0.001), IL‐10 (*p* = 0.004), IL‐1β, and TNF‐α (*p* < 0.001) levels at baseline. There were strong positive correlations between PGRN levels and PI (*p* < 0.001), GI (*p* = 0.001), BOP (%) (*p* = 0.005), PPD (*p* = 0.003), CAL (*p* = 0.002) values, and number of missing teeth (*p* = 0.002).

**TABLE 3:** Correlations between GCF PGRN total amount and GCF cytokines total amount and clinical periodontal parameters

Variables		VEGF	IL‐1β/IL‐1F2	TNF‐α	IL‐10	PI	GI	BOP (%)	PPD	CAL	No. of missing teeth
PGRN	r p	0.652 <0.001	0.675 <0.001	0.402 <0.001	0.464 0.004	0.583 <0.001	0.599 <0.001	0.612 <0.001	0.626 <0.001	0.630 <0.001	0.427 0.002

Abbreviations: BOP, bleeding on probing; CAL, clinical attachment level; GI, gingival index; IL, interleukin; PGRN, progranulin; PI, plaque index; PPD, probing pocket depth; TNF‐α, tumor necrosis factor‐alpha; VEGF, vascular endothelial growth factor.

*p*‐value was obtained from Spearman correlation test *r*: Spearman's rank correlation coefficient statistically significant at *p* < 0.05.

## 4 DISCUSSION

To the best of our knowledge, this is the first study to evaluate the effect of NSPT on GCF and serum PGRN levels and its correlation with proinflammatory and repair/regenerative mediators in the early healing phases. The findings of the present study show that GCF PGRN levels were higher in the test group than in the control group at baseline, and then decreased after NSPT on the 14th day in patients with periodontitis. Changes in PGRN in the early healing phase mirrored those of proinflammatory cytokines, and both pro‐inflammatory and regenerative mediators values decreased distinctly after NSPT on the 14th day. GCF PGRN/TNF‐α molar ratio levels decreased dramatically immediately after NSPT, with a subsequent increase at following timepoints, but remaining well below baseline levels.

Both total GCF levels per sampling time (30 s) and concentrations in 1 µL of GCF were calculated for the present study, as concentration values may be directly affected by the GCF volume, while total cytokine amounts in GCF samples per sampling time may be a better indicator of the relative activity of GCF components.^32^ Previous studies have reported that the total amount values of GCF are a more sensitive way of comparing GCF findings with concentration values.^32^, ^33^, ^34^ In this study, GCF volume was 10‐fold higher in periodontitis compared to controls, in agreement with previous studies.^32^, ^33^, ^34^ In the present study, all of the studied GCF mediators had higher values in the periodontitis group than controls at baseline, while for GCF concentration levels, this was the case only for TNF‐α and IL‐10.

The pattern of changes post‐NSPT showed an immediate short‐term increase for TNF‐α and IL‐10 and, to a smaller extent, for VEGF, while PGRN and IL‐1β/IL‐1F2 started decreasing post‐treatment. It is important to notice that the total amounts of all studied cytokines were lower at the last time‐point compared with baseline, compatibly with a reduction in total GCF levels. These findings were in line with previous studies including different time‐point evaluations and generally showing that VEGF levels increased in parallel with periodontal disease severity compared to periodontal health,^35^, ^36^ and an increase in VEGF levels after treatment in the early healing phase.^37^ The observed increase in TNF‐α levels on the 1st day post‐treatment is also consistent with the literature^38^ and in line with the early inflammatory phase, followed by a decrease in the later period,^37^, ^39^ while findings related to GCF TNF‐α concentrations post‐NSPT are less consistent.^37^, ^39^, ^40^ The similar pattern of short‐term post‐treatment increase in IL‐10 levels is also consistent with previous studies.^39^, ^41^ The decrease in IL‐1 β levels observed in the 1st and 3rd months healing period is also in keeping with previous literature.^36^, ^42^ In addition, the use of CHX mouthrinse and gel during the treatment of patients by full‐mouth disinfection may have an effect on the decrease in GCF mediators by contributing to the reduction of inflammation.^43^

The main novel finding of the study relates to PGRN, which is a protein with complex physiological functions; proteolytic cleavage produces granulin peptides that promote inflammatory activity, and the full‐length form of the protein has trophic and anti‐inflammatory activity.^44^ These bidirectional pro‐inflammatory or anti‐inflammatory functions play important roles in different diseases.^45^ The role of the PGRN has not been fully established in periodontal diseases. Hence, the present study elucidated the role of PGRN in the presence of periodontal disease and healing after NSPT. Few human studies have shown that PGRN levels increase in the presence of periodontitis.^18^, ^19^, ^20^ In line with these previous studies,^18^, ^19^, ^20^, ^46^, ^47^ the findings of the present study showed that GCF PGRN total amount levels were higher in the periodontitis group than in the periodontal healthy group. The increase in PGRN expression is mainly found in the extracellular matrix and cytoplasm of epithelial cells, and the upregulation of PGRN suggests that it may play a role in the natural response of gingival epithelial cells to inflammation.^18^ In addition, after the proteolytic cleavage of PGRN, small peptide fragments called granulins with proinflammatory properties are released.^19^ In the present study, the initial PGRN levels were similar to the high levels of proinflammatory mediators such as IL‐1β/IL‐1F2 and TNF‐α, suggesting that PGRN plays a role as a proinflammatory mediator.^19^, ^20^ However, the initial high values in VEGF and IL‐10 may indicate that regenerative mediators are elevated to tolerate inflammation. Granulin can stimulate the release of IL‐8 from epithelial cells and trigger a mechanism that acts as a chemoattractant for neutrophils.^45^ Therefore, evaluating the mediator levels in the wound healing period may give us a more accurate idea of how the disease affects the healing process. The present study evaluated PGRN levels at 5 different time points for wound healing after NSPT. GCF PGRN total amount levels decreased slightly after NSPT from the 1st day to the 14th day. Their level decreased significantly from the 14th day and reached the lowest level after 3 months. These findings are in line with a previous study^18^ which reported that GCF PGRN levels were higher in patients with periodontitis compared to healthy controls at baseline and decreased in the 1st month after treatment, and another study^45^ showed that PGRN saliva levels decreased 3 months after NSPT. The findings of the current study showed that after NSPT, in the early healing period, there is a decrease in this mediator on the 1st and 2nd days, although not statistically significant. On the other hand, the dramatic decrease seen from the 2nd day to the 14th day and continuing until the 3 months post‐treatment may be due to healing as a pro‐inflammatory mediator, and it can be interpreted as a decrease due to the reduction in inflammation, similar to regenerative mediators. The regenerative role of PGRN after periodontal treatment can be explained by its promotion of osteogenic differentiation in periodontal ligament stem cells and its support of bone homeostasis by inhibiting TNF‐induced osteoclastogenesis and promoting osteoblastogenesis.^17^, ^48^ However, this is only a speculation at this stage.

PGRN, as a TNF‐α antagonist inhibits TNF‐α–induced osteoclastogenesis and promotes osteoblastogenesis.^49^ PGRN/TNF‐α molar ratio may lead to understanding the role of PGRN in periodontal disease. In the current study, PGRN/TNF‐α molar ratios were similar in periodontitis patients and healthy controls. The ratios decreased immediately after treatment, remaining below levels throughout the study, due to the rapid increase in TNF levels on the 1st day after treatment, and then to the decrease in PGRN levels after day 14. Then, the ratio levels increased gradually from the 1st day to 1st month of the healing period. The previously mentioned study evaluating PGRN levels after periodontal treatment did not provide information about the PGRN/TNF molar ratio of healthy controls but the values increased at 1 month after treatment compared to the baseline.^18^ These data suggest that PGRN may indirectly affect the healing stages by an effect on TNF‐α, rather than directly. Also, the decrease in the PGRN/TNF ratio in the period after treatment compared to the active period of periodontal disease may be related to healing and resolution of inflammation after periodontal treatment. This may indicate that it increases as a defense mechanism during acute inflammation and decreases when inflammation is brought under control, thus approaching homeostasis.

PGRN levels had a positive correlation with VEGF, IL‐1β/IL‐1F2, TNF‐α, and IL‐10 levels in GCF. While all mediators showed a dramatic decrease due to decreased inflammation on day 14, the decrease in PGRN levels was parallel to IL‐1β/IL‐1F2 in the 1st and 2nd days of the early healing period. Additionally, there were positive correlations between PGRN and all clinical parameters. According to these findings, it can be suggested that PGRN can act as an inflammatory mediator.

The serum parameters did not appear different after NSPT in the present study; however, there was significant variation among individuals, even though the present study had a controlled study population. These results may indicate that periodontal disease and treatment have a limited effect on systemic mediators^50^ and serum levels of the mediators may be linked to the absence of a sufficiently severe local infection/inflammation within the oral cavity. The reasons for the significant variation among individuals can be the degree of local inflammation and the effect of biological individual factors, such as genetics.

The strengths of this study are the analysis of a panel of cytokines in periodontitis and healthy controls, as well as at several time points post‐treatment. A limitation of the study was that the values of the indicators were under the LOD for some mediators. Another limitation was that serum 3‐month values and the IL‐1β/IL‐1F2 and IL‐10 values could not be obtained, probably due to sample degradation.

## 5 CONCLUSION

Overall, this study contributes to our understanding of the cytokine dynamics following NSPT, with emphasis on PGRN, showing increased levels in disease and a decrease post‐treatment, in parallel with IL‐1β/IL‐1F2, while PGRN/ TNF‐α molar ratio levels increased gradually from the first day to the first month of the healing period. Examination of GCF mediators of repair/regeneration or inflammation at different time points following NSPT may facilitate the elucidation of periodontal wound healing processes and assist in the prediction of future periodontal treatment outcomes.

## AUTHOR CONTRIBUTIONS

Aysegul Sari and Luigi Nibali conceived the original idea. Aysegul Sari performed the methodology, statistical analysis, and writing of the original draft. Aysegul Sari and Pasquale Santamaria performed laboratory work. Luigi Nibali provided critical feedback and helped shape the manuscript.

## CONFLICT OF INTEREST STATEMENT

The authors declare no conflict of interest.

## ETHICS STATEMENT

The ethics approval with reference 2022/90 was granted by the Ethics Committee for the Use of Human Subjects in Research of Hatay Mustafa Kemal University.

## Supporting information



Supporting information

Supporting information

## Data Availability

All data sets used in this study are not publicly available due to ethical or legal restrictions. For inquiries regarding the data sets used in this study, please contact A.S., the principal investigator of this study, with reasonable requests.
