# Supplementary material for: The effect of non‐surgical periodontal treatment on progranulin levels
Source: J Periodontol. 2025 Aug 25;97(4):658–70. doi: 10.1002/jper.11396 (PMC13169471; doi:10.1002/jper.11396)
Supplement: Supplementary file 1 — Supporting information [file JPER-97-658-s002.docx]

**Supplementary Table 1:** Comparison of GCF biomarkers baseline and after treatment between groups

| **Variables** | **Periodontal health group (n=24)** | **Periodontitis group**  **(n=24)** | **P value***  **(between groups)** |
| --- | --- | --- | --- |
| **GCF PGRN total amount (pg/30)**  **Baseline**  **Day 1**  **Day 2**  **Day 14**  **Month 1**  **Month 3**  **P value** (among time points)** | 71.08 (36.28-91.91) | 435.379 (231.33-658.67)  409.37 (124.98-612.89)  322.22 (110.47-652.91)  99.5 (68.56-187.18) †, ‡, \|\|  123.78 (47.74-187.83) †, ‡, \|\|  37.22 (37.23-133.74) †, ‡, \|\|  <0.001 | <0.001 |
| **GCF PGRN concentration (pg/uL)**  **Baseline**  **Day 1**  **Day 2**  **Day 14**  **Month 1**  **Month 3**  **P value** (among time points)** | 284.54 (177.95-377.74) | 182.25 (111.82- 286.125)  181.99 (124.45-297.11)  178.22 (118.31-373.71)  209.19 (120.29-269.34)  213.21 (125.75-364.94)  248.19 (125.75-600.68)  0.598 | 0.073 |
| **GCF VEGF total amount (pg/30)**  **Baseline**  **Day 1**  **Day 2**  **Day 14**  **Month 1**  **Month 3**  **P value** (among time points)** | 2.32 (1.31-3.49) | 11.26 (8.71-18.33)  16.66 (11.53-19.19)  15.17 (8.72-20.53)  4.08 (2.78-5.56) †, ‡, \|\|  4.35 (3.21-7.61) †, ‡, \|\|  2.32 (1.13-5.04) †, ‡, \|\|  <0.001 | <0.001 |
| **GCF VEGF concentration (pg/uL)**  **Baseline**  **Day 1**  **Day 2**  **Day 14**  **Month 1**  **Month 3**  **P value** (among time points)** | 7.8 (3.52-15.86) | 4.09 (5.14-7.39)  6.69 (5.04-9.02)  8.7 (6.2-12.32) †  6.54 (4.09-11.79)  10.08 (13.67-7.0.1) †, ‡, §  8.81 (4.24-416.93) †  0.001 | 0.201 |
| **GCF IL-1β/IL-1F2 total amount (pg/30)**  **Baseline**  **Day 1**  **Day 2**  **Day 14**  **Month 1**  **Month 3**  **P value** (among time points)** | 11.29 (3.99-16.22) | 138.83 (77.51-218.64)  132.51 (97.67-222.07)  96.76 (52.28-145.81) ‡  20.14 (9.26-40.25) †, ‡, \|\|  21.82 (12.48-43.03) †, ‡, \|\|  7.93 (2.52-37.15) †, ‡, \|\|  <0.001 | <0.001 |
| **GCF / IL-1β/IL-1F2 concentration (pg/uL)**  **Baseline**  **Day 1**  **Day 2**  **Day 14**  **Month 1**  **Month 3**  **P value** (among time points)** | 51.56 (13.39-86) | 63.01 (43.01-93.84)  65.02 (42.19-119.45)  58.5 (34.61-76.57)  33.67 (16.28-56.17) †, ‡  46.85 (24.7-121.58) ‡  35.67 (11.11-105.06) †, ‡  0.012 | 0.108 |
| **GCF TNF-α total amount (pg/30)**  **Baseline**  **Day 1**  **Day 2**  **Day 14**  **Month 1**  **Month 3**  **P value** (among time points)** | 0.05 (0.02-0.08) | 0.27 (0.13-0.58)  1.35 (0.88-2.65) †  0.82 (0.41-1.93) †  0.14 (0.09-0.32) ‡, \|\|  0.19 (0.06-0.24) †, ‡, \|\|  0.21 (0.06-0.21) †, ‡, \|\|  <0.001 | <0.001 |
| **GCF TNF-α concentration (pg/uL)**  **Baseline**  **Day 1**  **Day 2**  **Day 14**  **Month 1**  **Month 3**  **P value** (among time points)** | 0.21 (0.11-0.41) | 0.06 (0.1-0.21)  0.76 (0.4-1.2) †  0.57 (0.36-1.03) †  0.3 (0.15-0.46) †, ‡, \|\|  0.41 (0.11-1.13) †, ‡, \|\|  0.64 (0.31-1.17) †, §  <0.001 | 0.039 |
| **GCF IL-10 total amount (pg/30)**  **Baseline**  **Day 1**  **Day 2**  **Day 14**  **Month 1**  **Month 3**  **P value** (among time points)** | 0.07 (0.06-0.09) | 0.11 (0.09-0.13)  0.11 (0.11-0.33) †  0.16 (0.14-0.31) †  0.16 (0.12-0.18) †  0.04 (0.02-0.09) ‡, \|\|  0.11 (0.03-0.14) ‡, \|\|, §  <0.001 | 0.002 |
| **GCF IL-10 concentration (pg/uL)**  **Baseline**  **Day 1**  **Day 2**  **Day 14**  **Month 1**  **Month 3**  **P value** (among time points)** | 0.18 (0.26-0.46) | 0.05 (0.03-0.07)  0.09 (0.05-0.15) †  0.13 (0.11-0.22) †  0.29 (1.15-0.39) †, ‡  0.09 (0.05-0.21) †, ‡, \|\|, §  1.11 (0.36-0.49) †, ‡, \|\|, §  <0.001 | <0.001 |
| **GCF PGRN/TNF-α molar ratio**  **Baseline**  **Day 1**  **Day 2**  **Day 14**  **Month 1**  **Month 3**  **P value** (among time points)** | 1159.49 (697.31-2876.94) | 2130.01 (711.69-3077.03)  289.39 (117.12-626.67) †  388.04 (255.26-482.33) †  712.68 (515.2-1071.18) †, ‡, \|\|  831.47 (248.35-1914.89) †, ‡, \|\|  186.41 (175.61-1464.78) †  <0.001 | 0.433 |
| **Serum PGRN concentration (pg/mL)**  **Baseline**  **Month 1**  **P value** (among time points)** | 14410.62 (11738.24-18975.07) | 16878.9 (14975.46- 19912.85)  16379.57 (15266.15-18218.93)  0.775 | 0.265 |
| **Serum VEGF concentration (pg/mL)**  **Baseline**  **Month 1**  **P value** (among time points)** | 22.39 (15.56-37.48) | 33.92 (22.84- 47.34)  38.8 (29.41-45.77)  0.597 | 0.058 |
| **Serum TNF-α concentration (pg/mL)**  **Baseline**  **Month 1**  **P value** (among time points)** | 1.28 (0.79-1.49) | 1.49 (0.89-1.92)  1.83 (1.44-2.21)  0.094 | 0.326 |
| **Serum PGRN/TNF-α**  **Baseline**  **Month 1**  **P value** (among time points)** | 14410.62 (117338.23-18975.06) | 11166.95 (8690.42-18251.46)  9333.71 (7280.97-12518.88)  0.063 | 0.117 |

*P value was obtained from Mann Whitney U test for between groups

**P value was obtained from the Friedman test for among times variables and Wilcoxon Signed-Rank Test for pairwise comparison.

Data are expressed as median and interquartile range (IQR: 25-75). Statistically significant at P< 0.05

† versus baseline, ‡ versus 24^th^ h, || versus 48^th^ h, § versus 14^th^ day, ¶ versus 1^st^ month

PGRN: Progranulin VGEF: Vascular endothelial growth factor IL-1β: Interleukin-1 beta TNF-α: Tumor necrosis factor-alpha IL-10: Interleukin-10
